# CD23 can negatively regulate B-cell receptor signaling

**DOI:** 10.1038/srep25629

**Published:** 2016-05-16

**Authors:** Chaohong Liu, Katharina Richard, Melvin Wiggins, Xiaoping Zhu, Daniel H. Conrad, Wenxia Song

**Affiliations:** 1 Department of Pathogen Biology, School of Basic Medicine, Huazhong University of Science and Technology, Wuhan 430030, China; 2 Department of Microbiology & Immunology, University of Maryland School of Medicine, Baltimore, MD 21201, USA; 3 Department of Cell Biology and Molecular Genetics, University of Maryland, College Park, MD 20742, USA; 4 Department of Veterinary Medicine, University of Maryland, College Park, MD 20742, USA; 5 Department of Microbiology and Immunology, Virginia Commonwealth University, Richmond, VA 23298, USA.

## Abstract

CD23 has been implicated as a negative regulator of IgE and IgG antibody responses. However, whether CD23 has any role in B-cell activation remains unclear. We examined the expression of CD23 in different subsets of peripheral B cells and the impact of CD23 expression on the early events of B-cell receptor (BCR) activation using CD23 knockout (KO) mice. We found that in addition to marginal zone B cells, mature follicular B cells significantly down regulate the surface expression level of CD23 after undergoing isotype switch and memory B-cell differentiation. Upon stimulation with membrane-associated antigen, CD23 KO causes significant increases in the area of B cells contacting the antigen-presenting membrane and the magnitude of BCR clustering. This enhanced cell spreading and BCR clustering is concurrent with increases in the levels of phosphorylation of tyrosine and Btk, as well as the levels of F-actin and phosphorylated Wiskott Aldrich syndrome protein, an actin nucleation promoting factor, in the contract zone of CD23 KO B cells. These results reveal a role of CD23 in the negative regulation of BCR signaling in the absence of IgE immune complex and suggest that CD23 down-regulates BCR signaling by influencing actin-mediated BCR clustering and B-cell morphological changes.

The CD23 molecule is an Fc receptor specific for IgE (FcεRII) that is expressed on the surface of B cells and follicular dendritic cells in mice and in variety of hematopoietic cells in humans, including B cells, T cell, follicular dendritic cells, macrophages, NK cells, eosinophils, and platelets^1^. As a member of the C-type lectin family, CD23 binds to IgE in a Ca^2+^ -dependent manner^2^,^3^. While CD23 was initially considered as a low affinity Fc receptor for IgE^4^, it was later found to have an affinity comparable to that of the high affinity IgE receptor, FcεRI, when forming oligomers^5^.

Although CD23 has been studied for more than two decades, its immunological function is not fully understood. Using CD23 knockout (KO) and transgenic mouse models, previous studies have revealed a complicated regulatory function of CD23 in the adaptive immune response. It is clear that the development of both B and T cells is independent of CD23 since their maturation is largely normal in both CD23 KO and transgenic mice^6^. CD23 has been shown to act as a negative regulator not only for IgE but also for IgG antibody responses in B cells^4^,^7^. This has been demonstrated with CD23 KO mice, which have greater levels of antigen-specific and total IgE and IgG in response to a protein antigen compared to those in wild type (wt) mice^8^. Conversely, the levels of both IgE and IgG antibody responses are significantly decreased in CD23 transgenic mice that over-expressed CD23, when compared to those in wt mice^7^. Using adaptive transfer approach, Payet-Jamroz *et al*. showed that the suppression of IgE, but not IgG responses, in CD23 transgenic mice is primarily dependent on the expression of CD23 in nonlymphoid cells, particularly follicular dendritic cells^9^. Furthermore, IgG B cells lacking CD23 differentiate into IgG-secreting cells much more efficiently than CD23 expressing IgG B cells *in vitro*^9^. These data suggest that CD23 can negatively regulate the activation and differentiation of both mIgE and mIgG expressing B cells into plasma cells, either by CD23-dependent interaction of B cells with other cells or by the interaction of CD23 with the BCR within B cells.

CD23 has been shown to be involved in B-cell activation *in vitro*. Colligation of the BCR with CD23 inhibits B-cell proliferative responses to BCR cross-linking activation via BCR^10^. Cross-linking of CD23 alone by IgE immune complexes or by anti-CD23 antibody also reduces the proliferation and plasma cell differentiation of B cells in response to BCR cross-linking and treatments of IL-4 or Staphylocuccus aureus Cowan I *in vitro*^11^. Furthermore, colligation of CD23 with the BCR leads to an elevation of cMyc and B-cell apoptosis^4^. These data support the notion that CD23 expressed on B cell negatively regulates BCR and B-cell activation. However, the molecular mechanisms underlying the inhibitory effect of CD23 on BCR activation is largely unknown.

BCR-mediated signaling is critical for the survival, activation and differentiation of B cells^12^. Recent studies using high resolution live cell imaging has revealed the early events of BCR activation at the B-cell surface^13^. The binding of the BCR to antigen induces rapid reorganization of surface BCRs into microclusters first and coalescence of BCR microclusters into central clusters later^14^. BCR clustering is concurrent with the morphological changes of B cells: spreading followed by contraction, when interacting with antigen presented on the cell membrane and immobile surface^14^. These early events depend on BCR proximal signaling and signaling-induced actin reorganization, and are regulated by the binding affinity of the BCR to antigen and the density of antigen on the presenting surface^15^. BCR signaling regulates the kinetics and extent of B-cell spreading and BCR clustering, which in turn influences the levels of BCR signaling and B-cell activation. The kinetics and magnitude of BCR clustering directly impact BCR signaling. BCR microclusters sequentially recruit key signaling mediators, including Lyn, Syk, PLCγ2 and Btk, which enable the formation of BCR microsignalosomes and the initiation of signaling cascades^16^. In contrast, BCR central clusters, the coalescent product of BCR microclusters, recruit the inhibitory phosphatase SHIP-1, leading to the down regulation of BCR signaling at the cell surface^17^.

Along with others, we have shown that actin remodeling is critical for the initiation and regulation of the early events of BCR signaling^17^,^18^ by controlling the lateral mobility of surface BCRs and B-cell morphology. Stabilization of the actin network inhibits the lateral movement and self-clustering of surface BCRs, consequently inhibiting signaling initiation^19^. Actin depolymerization increases BCR lateral mobility, which leads to BCR clustering and signaling activation in the absence of antigen. Actin-mediated B-cell spreading increases the contact of B cells with the antigen-presenting surface and enhances BCR clustering. B cell contraction promotes the coalescence of BCR microclusters into a central cluster, which leads to the attenuation of surface signaling^17^,^20^.

To understand the molecular mechanism by which CD23 negatively regulates BCR-mediated B-cell activation, we examined the relative expression levels of CD23 in peripheral B cells and the effects of CD23 KO on the early events of BCR activation in response to BCR cross-linking alone. We found that isotype switched B cells including memory B cells express a lower level of CD23 than naïve B cells. CD23 KO causes increases in the magnitudes of B cell spreading, actin accumulation, the protein tyrosine phosphorylation, as well as the phosphorylation of Btk and its downstream actin regulator, WASP, compared to those of WT B cells. These results indicate that CD23 can negatively regulate BCR signaling by promoting B-cell contraction and BCR central cluster formation and by modulating actin reorganization in the absence of CD23 cross-linking and colligation with the BCR.

## Materials and Methods

All the methods were carried out in accordance with the approved guidelines. All experiments involving mouse samples were performed using protocols approved by University of Maryland’s review board or animal care and usage committee and following University of Maryland’s as well as NIH guidelines and regulations.

### Mice and Cells

Wild type (BALB/c or C57BL/6) and CD23 knockout mice on BALB/C background of 6–10 weeks old were either purchased from Jackson Laboratories (Bar Harbor, ME) or kindly provided by Dr. Daniel Conrad at Virginia Commonwealth University^21^. To analyze CD23 expression levels in different B-cell subsets, C57BL/6 mice were immunized with 4-hydroxy-3-nitrophenylacetyl conjugated keyhole limpet hemocyanin (NP-KLH, 200 μg/mouse) twice 4 weeks apart and euthanized at 100 days post the first immunization. Splenic B cells were isolated as previously described^17^.

### Flow cytometry

The surface expression level of CD23 on splenic B cells was analyzed by flow cytometry. B cells were pre-incubated with anti-CD16/CD32 antibody (BD Biosciences, San Jose, CA, USA, Cat. No 553142) to block FcγR, and following by PerCP-Cy5.5-anti-B220 (BD Biosciences, Cat. No 552771), PE-anti-CD138 (BD Biosciences, Cat. No 553714), PE-Cy7-anti-IgM (BD Biosciences, Cat. No 552867), biotin-anti-IgD (Southern Biotech, Birmingham, AL, USA, Cat. No 1120-08), FITC-anti-IgD (BD Biosciences, Cat. No 553439), FITC-anti-IgM (BD Biosciences, Cat. No 553408) and APC-NP_19_, and FITC-anti-CD23 antibodies (BD Biosciences, Cat. No 553138), plus AF405- streptavidin (Thermo Scientfic, Waltham, MA, USA ,Cat. No S32351). After washing and fixation, cells were analyzed using a flow cytometer (BD CantoII, BD Biosciences). Data were analyzed using FACSDiva (BD Biosciences) and FlowJo (Treestar Inc. Ashland, OR, USA) software.

### Total Internal Reflection fluorescence Microcopy and image analysis

The surface distribution of the BCR and other molecules were analyzed using a TIRFm (TE2000U, Nikon, Melville, NY, USA). To image intracellular molecules, B cells were incubated with Alexa Fluor 546-conjugated monobiotinylated Fab’ fragment of anti-mouse IgG + M (AF546-mB-Fab’-anti-Ig) tethered lipid bilayers^17^ at 37 °C for varying lengths of time. Cells were fixed with 4% paraformaldehyde, permeabilized with 0.05% saponin, and stained for phosphotyrosine (pY) (Millipore, Billerica, MA, USA, Cat. No 05–321), phosphorylated Btk (pBtk, Y551; BD Biosciences, Cat. No 558034) and WASP (pWASP, S483/S484; Bethyl Laboratory, Montgomery, TX ,USA, Cat. No A300-205A), as well as for F-actin by AF488-phalloidin (Thermo Scientfic, Cat. No A12379). GFP/AF488, AF546, and interference refection images (IRM) were acquired sequentially at each time point. B-cell contact area was determined using TIRFm images and MATLAB software. Total (TFI) and mean fluorescence intensities (MFI) of AF546-mB-Fab’-anti-Ig in the contact zone were quantified using Andor iQ software (Andor Technology, Belfast, UK). Background fluorescence was subtracted. For each set of data with statistics, more than 60 individual cells from two or three independent experiments were analyzed.

### RT-PCR

Splenic B cells from WT and CD23 KO mice were sorted with flow cytometry by PerCP-Cy5.5-anti-B220. Total RNA was extracted with RNAPURE kit (RP1202; BioTeke, Beijing, China) and reverse-transcribed with a PrimeScript™ RT reagent Kit (RR037A; Takara, Dalian, China). The resulting cDNA was analyzed for the expression of various genes with SsoAdvancedTM SYBR® Green supermix (Bio-Rad, Hercules, CA, US) on a CFX96 Touch Real-Time System (Bio-Rad) and the appropriate primers for ‘test genes’. *cd 23* 5′ primer: cccaatcccagaactcaaaa, *cd 23* 3′ primer : ggaaatggagccagttcttg.

### Phos flow

Splenic B cells from WT and CD23 KO mice were incubated with monobiotinylated Fab’ fragment of anti-mouse IgG + M (mB-Fab’-anti-Ig) plus streptavidin at 37 °C for varying lengths of time^19^. Cells were fixed with Phosflow Lyse/Fix buffer, followed by permeabilization with Phosflow Perm buffer III (BD Biosciences, Cat. No. 558050) and staining with the following antibodies: PE-anti-Erk (T202/Y204, BD Biosciences, Cat. No 612566), AF647-anti-Akt (S473, BD Biosciences, Cat. No 561670) and PE-anti-Btk (Y551, BD Biosciences, Cat. No 558129).

### Statistics

The significance of differences between two sets of data was determined using two tailed student *t* test.

## Results

### Isotype switched and memory B cells down-regulate CD23 expression

**Figure 1:**
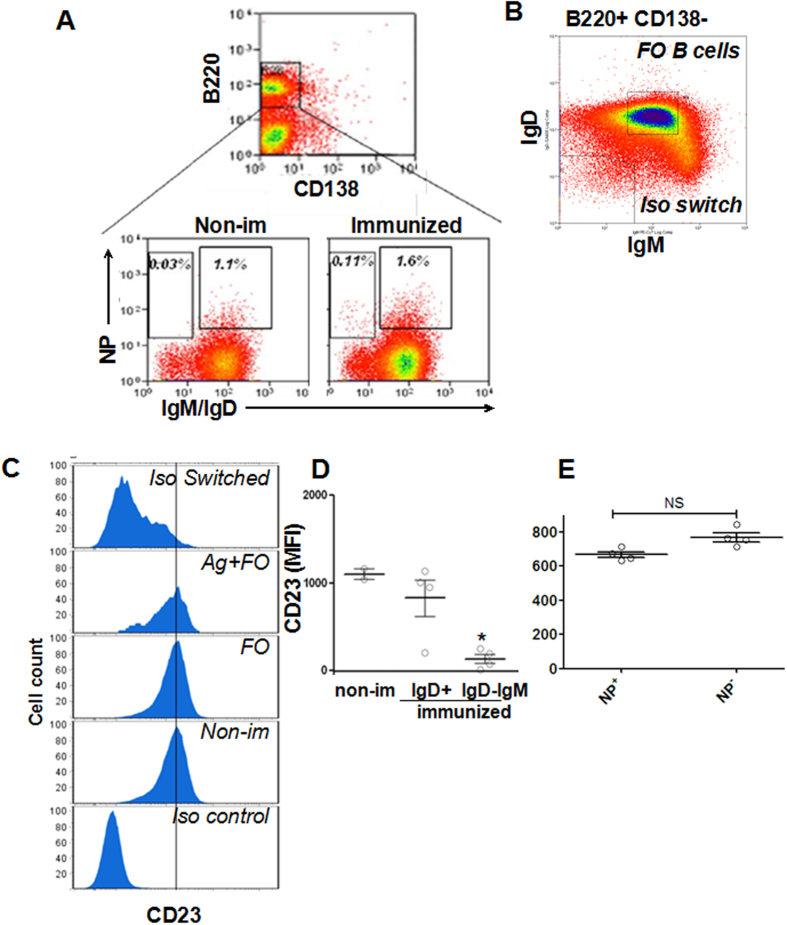
The expression levels of CD23 are reduced in isotype switched and memory B cells compared to follicular unswitched B cells. The expression levels of CD23 in indicated B-cell subsets from a representative mouse 100 days post first immunization were determined using flow cytometry. Splenic B cells were isolated from non-immunized mice and mice 100 days post the first immunization with NP-KLH and Ribi adjuvant. Cells were stained with FITC-anti-IgD, FITC-anti-IgM, PE-anti-CD138, PerCP-Cy5.5-anti-B220 Abs and allophycocyanin-NP_19_ (**A**). B-cell subsets were identified by specific surface markers: NP-binding memory B cells (Memory) B220^+^ CD138^−^ IgM^−^ IgD^−^ NP^+^, NP-binding unswitched follicular B cells (Ag^+^ FO) B220^+^ CD138^−^ IgM^int^ IgD^+^ NP^+^, naïve follicular B cells (FO) B220^+^ CD138^−^ IgM^int^ IgD^+^ NP^−^ (**A,B**). Shown are representative histograms (**C**), the dot blot to show the gating stretagy(A and B), and the average (±SD) of the mean fluorescence intensity (MFI) of CD23 in follicular B cells from non-immunized mice (non-im), and follicular isotype unswitched (IgD^+^) and switched (IgD^−^IgM^−^) B cells (**D**), and NP^+^ or NP^−^ B cells (**E**) from immunized mice. n = 4. **p < 0.01.

To investigate whether CD23 has any role in B-cell activation, we determined the expression levels of CD23 in different subsets of B2 B cells, as it is well known that marginal zone B cells express a much lower level of CD23 than B2 B cells. To generate memory B cells, we immunized mice with 4-hydroxy-3-nitrophenylacetyl-conjugated keyhole limpet hemocyanin (NP-KLH). We identified different B-cell subsets using their surface markers, including antigen-specific memory B cells (B220^+^ IgD^−^IgM^−^NP^+^) (Fig. 1A), follicular B cells (B220^+^ IgD^h^IgM^Int^), and isotype switched B cells (B220^+^ IgD^−^IgM^−^) (Fig. 1B). We have previously shown that cells with the phenotype of B220^+^ IgD^−^IgM^−^NP^+^ isolated from immunized mice 100 days post the immunization contain memory B cell properties^22^. By gating different subsets of B cells, we found the surface expression levels of CD23 in memory and isotype switched B cells from immunized mice was significantly lower than follicular B cells, despite if they were NP positive or not and they were from immunized or non-immunized mice or not (Fig. 1C,D). Furthermore we analyzed the CD23 expression in NP^+^ and NP^−^ B cell subsets and found that the levels of CD23 expression did not differ between NP^−^ B cells and NP^+^ B cells, which indicates the down regulation of CD23 is irrelevant for antigen specificity (Fig. 1E). Taken together, these results suggest that follicular B cells down-regulate CD23 expression after undergoing isotype switching and differentiation into memory B cells.

### CD23 deficiency impacts the early cellular event of BCR activation

**Figure 2:**
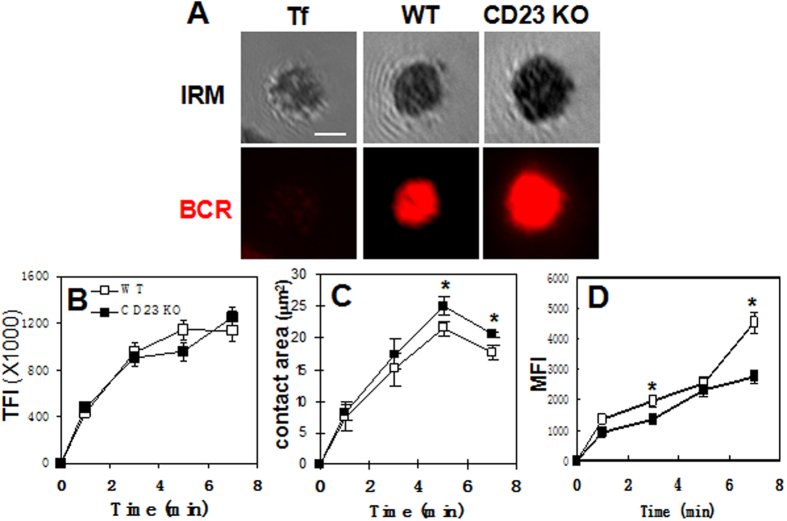
Effects of CD23 gene deletion on B-cell morphology and BCR clustering. Splenic B cells from WT and CD23 KO mice were incubated with AF546-mB-Fab’-anti-Ig tethered lipid bilayers at 37 °C. As a non-antigen control, WT B cells were labeled with AF546-Fab’-anti-Ig for the BCR before incubation with biotinylated transferrin (Tf) tethered to lipid bilayers. The B-cell contact area and the fluorescence intensity of AF546-mB-Fab’-anti-Ig in the contact zone were quantified. Shown are representative images of cells at 7 min (**A**) and the average values (±SD) of the total (TFI) (**B**) and the contact area (**C**) and mean fluorescence intensity (MFI) of AF546-mB-Fab’-anti-Ig in the contact zone (**D**) from 50 cells of three independent experiments. Scale bar, 2.5 μm. *p < 0.05.

In order to examine the role of CD23 in BCR activation, we determined the effect of CD23 deficiency on BCR clustering and B-cell spreading by using CD23 KO mice. We examined the deletion efficiency of CD23 KO mice by using flow cytometry and real-time PCR. We found both the protein expression levels and mRNA levels of CD23 were significantly reduced in CD23 KO B cells compared to that of wt B cells (Fig. S1A,S1B). These results suggest that CD23 was sucessfully knocked out in CD23 KO mice. BCR clustering and B-cell spreading were determined by the total fluorescence intensity (TFI) or MFI and the contact area between B cells and contacting antigens. In order to exclude the influence of different size between wt and CD23 KO B cells on the cell spreading, we examined the relative cell size of CD23 KO B cells by determing the FSC ratio of CD23 KO B cells with wt B cells. We did not find that the cell size differed between WT and CD23 KO B cells (Fig. S1C). Splenic B cells from wt and CD23 KO mice were stimulated with AF546-mB-Fab’-anti-Ig-tethered lipid bilayers. Transferrin tethered to lipid bilayers was used as a non-antigenic control. Similar to what we previously showed^17^, surface BCRs in wt B cells interacting with mB-Fab’-anti-Ig but not transferrin-tethered lipid bilayers clustered rapidly. The total fluorescence intensity (TFI) of AF546-mB-Fab’-anti-Ig plateaued by ~5 min (Fig. 2A,B) when the area of the B-cell membrane region contacting mB-Fab’-anti-Ig (B-cell contact zone) became maximal (Fig. 2A,C). The mean fluorescence intensity (MFI) of AF546-mB-Fab’-anti-Ig in the contact zone (Fig. 2D) continuously increased even when the TFI reached plateau (Fig. 2B) since the B-cell contact area decreased at 7 min indicating B cell-contraction (Fig. 2C). While the increasing kinetics and magnitudes of the AF546-mB-Fab’-anti-Ig TFI in the contact zone of CD23 KO B cells were similar to those of wt B cells (Fig. 2B), the MFI of AF546-mB-Fab’-anti-Ig in the contact zone of CD23 KO B cells decreased (Fig. 2D) and the contact area of CD23 KO B cells increased (Fig. 2C), compared to wt B cells. These results indicate that CD23 can regulate BCR activation by modulating the early cellular events, even though its effects are modest, and that CD23 promotes B-cell contraction, facilitating coalescence of BCR microclusters into central clusters, a process that has been shown to be related to signal down-regulation^17^,^20^.

### CD23 KO causes increases in BCR signaling at the B-cell surface

**Figure 3:**
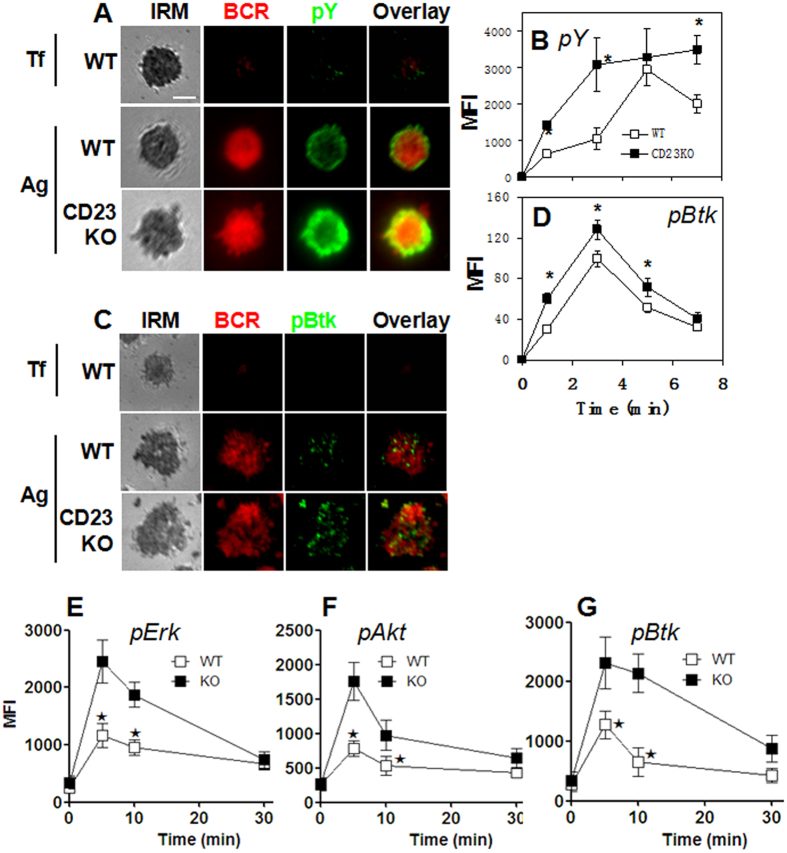
CD23 deficiency causes increases in BCR signaling at the B-cell surface. Splenic B cells from WT and CD23 KO mice were incubated with AF546-B-Fab’-anti-Ig tethered to lipid bilayers at 37 °C for indicated times. Cells were fixed, permeabilized, and stained for phosphotyrosine (pY) (**A,B**) and phosphorylated Btk (pBtk) (**C,D**). Cells were analyzed by TIRFm. Shown are representative images of cells at 7 min (**A,C**). The average MFI (±SD) were determined from 70 cells of three independent experiments (**B,D**). Splenic B cells from wt and CD23 KO mice were incubated with mB-Fab’-anti-Ig plus streptavidin at 37 °C for indicated times. Cells were fixed, permeabilized, and stained for pErk (**E**), pAkt (**F**) and pBtk (**G**). Cells were analyzed by flow cytometry. Scale bar, 2.5 μm. *p < 0.05.

BCR clustering and B cell spreading are required for the initiation and amplification of BCR signaling. The effects of CD23 deficiency on BCR clustering and B cell spreading potential lead to changes in BCR signaling. To test this possibility, we evaluated BCR signaling by measuring the level of phosphotyrosine (pY) and phosphorylated Btk (pBtk). The MFI of pY in the contact zone of wt B cells increased during the first 5 min of activation and decreased at 7 min when B cells contracted upon antigen stimulation (Fig. 3A,B). However there was no detection of the acumulation of pY in the contact zone of wt B cells upon non-antigenic stimulation with Tf, which indicates that the recruitment of pY in the contact zone is an antigen specific event (Fig. 3A,B). The pY MFI in the contact zone of CD23 KO B cells rapidly increased during the first 3 min of stimulation and remained at the maximal level up to 7 min, showing no sign of decreasing (Fig. 3A,B). Consequently, the level of pY in the contact zone of CD23 KO B cells was significant higher than that in WT B cells (Fig. 3B), even though the pY staining was mainly concentrated at the outer edge of the BCR central cluster at 7 min in both wt and CD23 KO B cells (Fig. 3A). Furthermore, the MFI of pBtk in the contact zone of CD23 KO B cells was significantly higher that that in wt B cells, even through the pBtk MFI rose and fell in similar timings and pBtk staining showed similar distribution in both wt and CD23 KO B cells (Fig. 3C,D). We also can not detect the recruitment of pBtk in the contact zone of wt B cells upon non-antigenic stimulation with Tf (Fig. 3C). In order to further confirm that CD23 negatively regulates BCR signaling, we examined the levels of phosphorylated Erk , Akt and Btk upon soluble antigen stimulation by using phos flow. Consisent with the results of B cells stimulated by mAgs, the levels of pErk, pAkt and pBtk were all enhanced in CD23 KO B cells upon soluble antigen stimulation compared to that of wt B cells (Fig. 3E–G). There results indicate that CD23 can negatively regulate BCR signaling induced by both membrane-associated antigen and soluble antigen.

### CD23 KO leads to increases in F-actin accumulation and WASP activation at the surface of B cells

**Figure 4:**
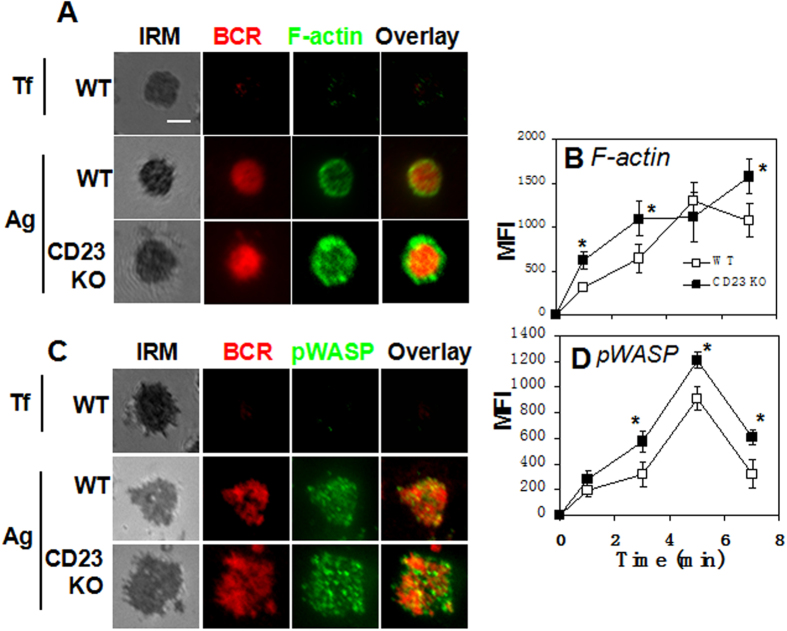
The levels of F-actin accumulation and phosphorylated WASP increase in the activation sites of CD23 KO B cells. Splenic B cells from WT and CD23 KO mice were incubated with AF546-mB-Fab’-anti-Ig tethered to lipid bilayers at 37 °C for indicated times. Cells were fixed, permeabilized, and stained for F-actin and phosphorylated WASP (pWASP). Cells were analyzed using TIRFm. Shown are representative images of cells at 7 min (**A,C**) and the average MFI (±SD) in the B-cell contact zone (**B,D**) determined from 80 cells of three independent experiments. Scale bar, 2.5 μm. *p < 0.05.

Antigen-induce BCR clustering and B cell morphological changes depend on actin remodeling. The effects of CD23 on BCR clustering and B-cell spreading suggest an involvement of CD23 in actin remodeling. In order to investigate this hypothesis, we examined the effect of CD23 deficiency on actin accumulation and the activation of the actin nucleation promoting factor- WASP at the B-cell contact zone induced by mB-Fab’-anti-Ig-tethered lipid bilayer. F-actin was labeled with AF488-phalloidin and active WASP by antibody specific for phosphorylated WASP (pWASP). As shown in our previously published studies^17^,^19^, the MFI of F-actin in the contact zone of wt B cells rapidly increased as B cells spread, peaked at 5 min as the B-cell contact area became maximal, and decreased at 7 min when B cells contracted (Fig. 4A,B). There was no dectection of the accumulation of F-actin in the contact zone of wt B cells upon non-antigenic stimulation with Tf (Fig. 4A). Compared to wt B cells, the MFI of F-actin in the contact zone of CD23 KO B cells was significant higher and continuously increased up to 7 min, showing no sign of decreasing (Fig. 4B). However, the distribution pattern of F-actin in the contact zone of wt and CD23 KO B cells appeared to be similar, showing F-actin accumulation at the outer edge of the central BCR cluster at 7 min (Fig. 4A). Consistent with an increase in F-actin accumulation, the MFI of pWASP in the contact zone of CD23 KO B cells was significantly higher than that in wt B cells (Fig. 4C,D). The accumulation of pWASP is not detectable in the contact zone of wt B cells upon non-antigenic stimulation with Tf (Fig. 4C). However, CD23 deficiency did not cause any changes in timings for the rising and fall of pWASP levels and the distribution pattern of pWASP in the B-cell contact zone (Fig. 4C,D). These data indicate that CD23 is involved in regulating actin remodeling during BCR activation particularly in the stage of actin clearance, and also suggest that modulating the activity of WASP and/or signaling molecules upstream of WASP is a potential mechanism underlying CD23’s roles in actin remodeling.

## Discussion

This study reveals a role for CD23 in the negative regulation of BCR signaling induced by stimulation of membrane-associated antigen and soluble antigen. Since the inhibitory effect of CD23 was observed in the absence of IgE-immune complexes, this indicates that CD23 can exert its negative regulatory function without being crosslinked and colligated with the BCR. This provides a potential explanation for the inhibitory effect of over expression of CD23, an Fc receptor for IgE, on IgG antibody responses, since the binding of CD23 to IgE-immune complexes is not absolutely required for its inhibitory role. However, it is notable that the effects of CD23 KO on BCR signaling induced by BCR engagement with antigen are moderate at most, which may be the reason why the inhibitory effect of CD23 expression on IgG antibody responses has not been consistently detected^6^.

We have revealed here that in addition to marginal zone B cells, which express a much lower level of CD23 on their surface than that of follicular B cells, antigen-specific memory B cells and isotype switched B cells originated from follicular B cells significantly down-regulate CD23 expression levels. This down-regulation would reduce or eliminate the inhibitory effects of CD23 on BCR signaling, and allow these B cells to mount higher levels of BCR signaling and B-cell activation than follicular B cells and to decrease their sensitivity to IgE-immune complexes. Therefore, the down-regulation of CD23 potentially is one of the mechanisms that enable memory B cells to mount fast and robotic clonal expansion and antibody responses in response to antigenic stimulation. However, memory B cells from schistosome-infected children show higher expression of CD23 upon stimulation, but lower proliferation and TNF-α production^23^. This may be due to the differences of mice and human or NP-KLH immunization may not appropriately mimic parasitic infection. While CD23 expression is known to be induced and enhanced by inflammatory cytokines^24^, the external and internal signals and upstream signaling mechanisms that lead to the reduction in the surface expression of CD23 in isotype switched and memory B cells are largely unknown.

The results from this study further show that the regulatory effect of CD23 on BCR signaling at least in part is mediated by direct and indirect influences of surface CD23 molecules on actin-dependent BCR clustering and B-cell morphological changes. Upon interacting with membrane-associated antigen, surface BCRs reorganize into microclusters, where BCRs interact with lipid rafts and signaling molecules and initiate signaling cascades^25^. Actin-mediated B-cell spreading enhances this processing^15^. We have recently demonstrated that signaling attenuation is associated with the clearance of the actin cytoskeleton from the B-cell contact zone and actin-driven B-cell contraction and coalescence of BCR microclusters into polarized central clusters^17^. Here we show that CD23^−/−^ B cells fail or delay the actin clearance and B-cell contraction, consequently interfering with the coalescence of BCR microclusters that is required for signaling attenuation. The molecular mechanisms by which CD23 regulates these actin-dependent events are unknown. The increased levels of both phosphorylated Btk and WASP in CD23^−/−^ B cells showed here suggest that CD23 can suppress actin dynamics by inhibiting Btk, as Btk can activate WASP by inducing WASP phosphorylation and by activating the guanine nucleotide exchange factor of Cdc42 and the production of phosphatidylinositol lipids^26^. Btk activation is induced by BCR crosslinking and can be enhanced by the stimulatory coreceptor complex CD19/CD21/CD81^27^. Co-crosslinking CD23 with BCR leads to a reduction in the basal levels of phosphorylation of Btk^28^. CD23 may be able to directly interfere with Btk activation through interacting with CD21, as reported previously^27^.

In summary, the data presented here demonstrate a negatively regulatory function of CD23 in the activation of B cells stimulated by antigen alone. CD23 suppresses BCR signaling via promoting B-cell contraction and coalescence of BCR microclusters into central clusters, cellular events that associate with signaling attenuation. We reveal that memory B cells down-regulate the expression level of CD23, which potentially contributes to higher levels of responses by memory B cells to antigenic stimulation than follicular B cells.

## Additional Information

**How to cite this article**: Liu, C. *et al*. CD23 can negatively regulate B-cell receptor signaling. *Sci. Rep*. **6**, 25629; doi: 10.1038/srep25629 (2016).

## Supplementary Material

Supplementary Information
