# Supplementary material for: CD23 can negatively regulate B-cell receptor signaling
Source: Sci Rep. 2016 May 16;6:25629. doi: 10.1038/srep25629 (PMC4867583; doi:10.1038/srep25629)
Supplement: Supplementary Information [file srep25629-s1.doc]

CD23 can negatively regulate B-cell receptor signaling

Chaohong Liu^1﹡^, Katharina Richard^3^, Melvin Wiggins^2^, Xiaoping Zhu^4^, Daniel H. Conrad^5^, and Wenxia Song^2﹡^

^1^Department of Pathogen Biology, School of Basic Medicine, Huazhong University of Science and Technology, Wuhan 430030, China

^2^Department of Cell Biology and Molecular Genetics, University of Maryland, College Park, MD 20742, USA

^3^Department of Microbiology & Immunology, University of Maryland School of Medicine, Baltimore, MD 21201, USA

^4^Department of Veterinary Medicine, University of Maryland, College Park, MD 20742

^5^Department of Microbiology and Immunology, Virginia Commonwealth University, Richmond, VA 23298


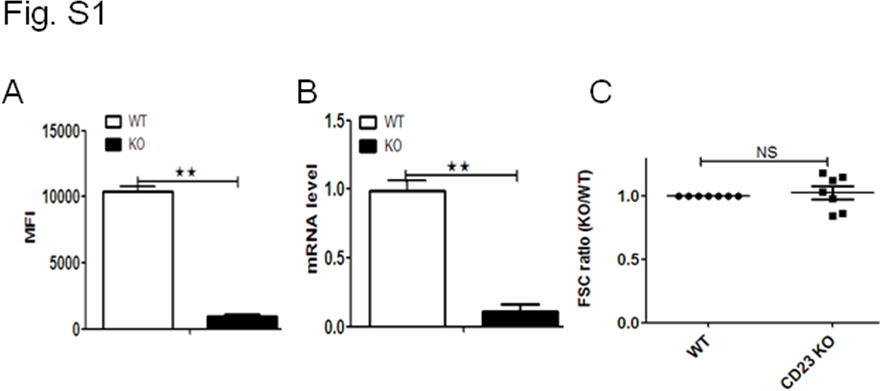


Figure S1. The mRNA and protein expression levels of CD23 are significantly decreased in CD23 KO mice

Splenic B cells from wt and CD23 KO mice were incubated with FITC-anti-CD23 and PerCP-Cy5.5-anti-B220 for 30 min on ice followed by flow cytomery (A). RNAs were extracted from splenic B cells of wt and CD23 KO mice sorted by PerCP-Cy5.5-anti-B220 antibodies, followed by real-time PCR with specific primers for *cd23* (B). The quantification of FSC values of wt and CD23 KO B cells by using flow cytometry.
